# The Specific Sagittal Magnetic Resonance Imaging of Intradural Extra-Arachnoid Lumbar Disc Herniation

**DOI:** 10.1155/2012/383451

**Published:** 2012-02-16

**Authors:** Tatsuro Sasaji, Kiyoshi Horaguchi, Noboru Yamada, Kazuo Iwai

**Affiliations:** Department of Orthopedic Surgery, Fukushima Rosai Hospital, 3-Numajiri, Tsuzura-machi, Uchigo, Iwaki 973-8403, Japan

## Abstract

Intradural extra-arachnoid lumbar disc herniation is a rare disease. Few MRI findings have been reported. We experienced an intradural extra-arachnoid lumbar disc herniation. We reviewed the preoperative MRI findings. Lumbar spine T2-weighted sagittal MRI showed that one line of the ventral dura was divided into two by a disc herniation. We speculated that the two lines comprised the dura and arachnoid and that a disc herniation existed between them. We believe that division of the ventral dural line on T2-weighted sagittal images is a characteristic finding of intradural extra-arachnoid lumbar disc herniation. The division of ventral dural line seemed to be a “Y,” and, thus, we called it the “Y sign.” The “Y sign” may be useful for diagnosing intradural extra-arachnoid lumbar disc herniation.

## 1. Introduction

Intradural extra-arachnoid lumbar disc herniation is a rare disease. Because of its rarity, few preoperative magnetic resonance imaging (MRI) studies have been reported, thus making a preoperative diagnosis difficult. Subdural space leakage of contrast medium by discography is an important criterion for intradural extra-arachnoid disc herniation [1]. It is impossible to perform discography for all lumbar disc herniation cases, and no studies have reported specific MRI findings for discography. We experienced an intradural extra-arachnoid lumbar disc herniation case; here we report the MRI findings.

The patient was informed that his data would be submitted for publication, and he gave us his consent.

## 2. Case Report

A 56-year-old male was suffering from right thigh pain, numbness, and gait disturbance for 1 month before presentation.

### 2.1. Neurological Examination

Neurological examination showed that his muscle power was normal in lower extremities and his right patellar tendon reflex was absent. Sensory disturbance was detected in the right thigh. The patient was diagnosed with right L3 radiculopathy. All laboratory findings were within normal limits.

### 2.2. Radiological Findings

**Figure 1:**
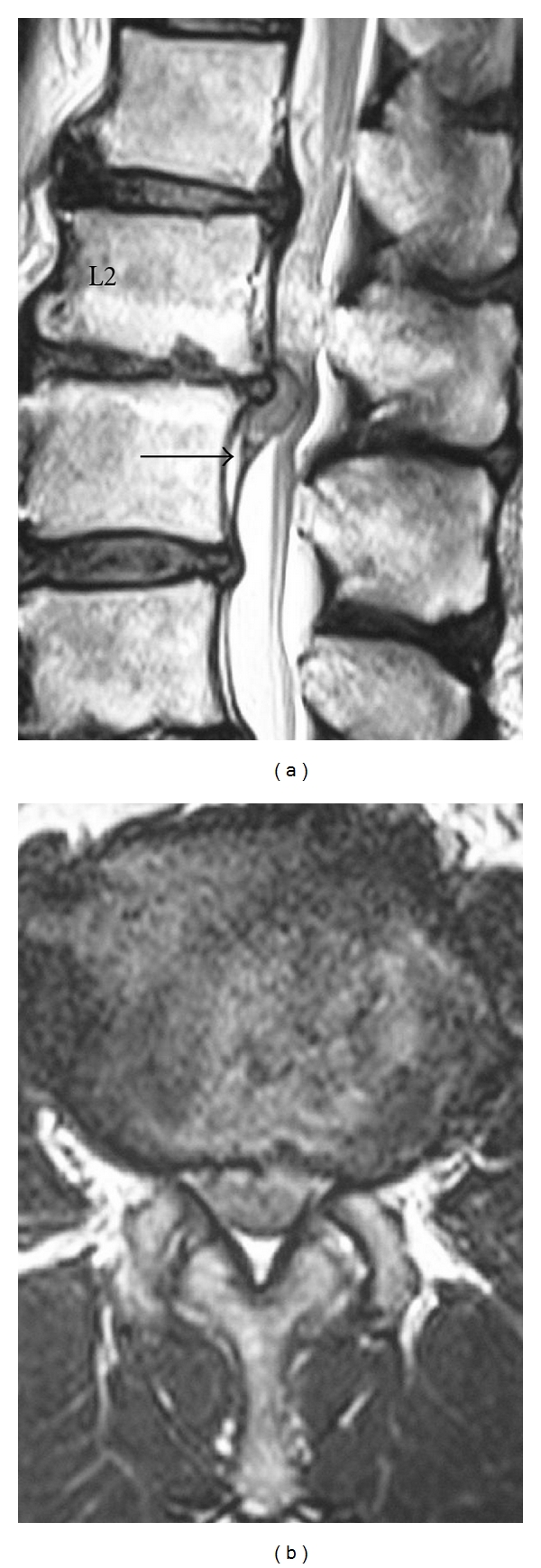
Preoperative MRI of the lumbar spine: (a) sagittal plane T2-weighted image. (b) Axial plane T2-weighted image. The ventral dural line was divided such that disc herniation existed between the two lines (black arrow) (a). The border of cauda equina and disc herniation was unclear (b).

Lumbar spine MRI showed a mass compressing the dura that caudally migrated at the L2-L3 level on T2-weighted sagittal image (Figure 1(a)). The mass continued to the disc and was assumed to be a lumbar disc herniation. The ventral dural line was divided into two near the disc herniation such that the herniation existed between these lines. The cauda equina was compressed by the disc herniation on T2-weighted axial images (Figure 1(b)). Intradural extra-arachnoid disc herniation was diagnosed.

### 2.3. Operation

**Figure 2:**
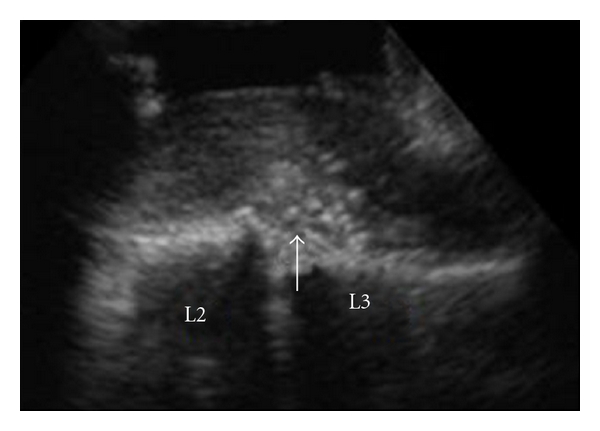
Intraoperative ultrasonography. Disc herniation was discovered in the dura (white arrow).

**Figure 3:**
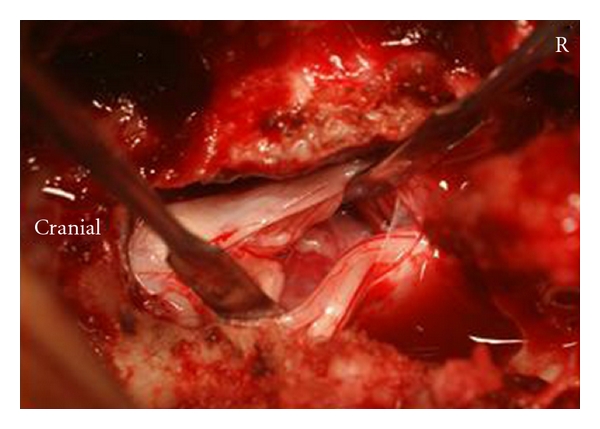
Intraoperative photograph. We identified the lumbar disc herniation covered with the arachnoid.

The lumbar spine was explored through a straight posterior midline approach. Partial laminectomy at L2-L3 revealed no disc herniation in the epidural space. We identified a disc herniation in the dura with an ultrasonography (Figure 2). Durotomy revealed fragments covered with the arachnoid (Figure 3). After removing the fragments, we found a hole in the ventral aspect of the dura; this hole was connected to the disc space. We did not suture the ventral dural defect to avoid the risk that threads irritate cauda equina.

### 2.4. Postoperative Course

The patient's leg symptom disappeared soon after surgery. One year after surgery, he has returned to a normal daily life with moderate low back pain.

## 3. Discussion

Dandy reported intradural lumbar disc herniation for the first time; the incidence of intradural lumbar disc herniation was between 0.04 and 0.33% [2, 3]. Because of rarity, the difference in radiological findings between intradural lumbar disc herniation and typical extradural lumbar disc herniation has been unclear. In some reports, the diagnosis was confirmed intraoperatively [4–6]. The preoperative diagnosis of intradural lumbar disc herniation has been difficult. We thought that the characteristic radiological findings of intradural lumbar disc herniation were necessary.

**Figure 4:**
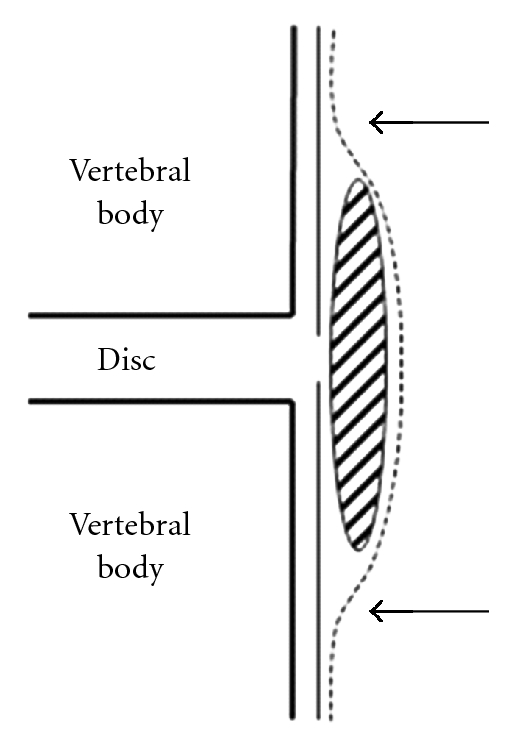
Schema for intradural extra-arachnoid lumbar disc herniation. An intradural extra-arachnoid lumbar disc herniation existed between the dura and arachnoid. The division of dura and arachnoid appeared as “Y” (shaded area: disc herniation; solid line: dura; dotted line: arachnoid; black arrow: “Y sign”).

Choi et al. reported useful MRI findings such as the “hawk-beak sign” and abrupt loss of posterior longitudinal ligament continuity [7]. The “hawk-beak sign” was a sharp compressing lesion with a beak-like appearance to the dural sac on T2-weighted axial images. However, these findings may not be specific to intradural lumbar disc herniation and were not observed in our case. According to Whittaker and D'Andrea et al., MRI with gadolinium was useful for diagnosis [8, 9]; however, contrast-enhanced MRI may be impossible in all lumbar disc herniation cases. Intradural extra-arachnoid disc herniation occurs between the dura and arachnoid (Figure 4). In the case of intradural extra-arachnoid disc herniation, the arachnoid was peeled from the dura by the disc herniation. One line of the dura and arachnoid was divided into two lines of the dura and the arachnoid. The branch of the ventral dural line appeared as “Y.” This branch may have been a characteristic appearance of intradural extra-arachnoid lumbar disc herniation, and, thus, we called it the “Y sign.” To our knowledge, such findings have not been reported previously.
